# Simultaneous Occurrence of Wilson’s Disease, Autoimmune Hepatitis, and Hereditary Hemochromatosis: A Diagnostic Challenge

**DOI:** 10.34172/mejdd.2024.371

**Published:** 2024-01-31

**Authors:** Reza Fatemi, Shahryar Movassagh-koolankuh, Nazanin Mosadeghi

**Affiliations:** ^1^Research Institute for Gastroenterology and Liver Diseases, Shahid Beheshti University of Medical Sciences, Tehran, Iran

**Keywords:** Autoimmune hepatitis, Hereditary hemochromatosis, Wilson’s disease, Acute liver failure

## Abstract

This is not surprising to detect iron overload in chronic liver diseases and end-stage liver diseases since Kupffer cells scavenge necrotic hepatocytes during the course of liver damage, leading to an increased serum iron level and transferrin saturation compatible with iron overload even in the absence of a genetic mutation suggestive of hereditary hemochromatosis. Therewith, a relative association has been found between some sorts of chronic liver diseases like non-alcoholic steatohepatitis and hepatitis C with human homeostatic iron regulator protein (HFE: High Fe^2+^) gene mutations. Moreover, impairment of ceruloplasmin ferroxidase activity in the course of Wilson’s disease (WD), leading to the accumulation of ferrous ions just like what is expected in aceruloplasminemia, is another known reason for iron overload accompanied by chronic liver disease. Of chronic liver diseases, autoimmune hepatitis (AIH), and cholestatic liver diseases are less related to iron overload. Accordingly, the coexistence of WD, AIH, and hereditary hemochromatosis when there exist clinical features, laboratory tests, genetic confirmation, and histological evaluations indicative of the three mentioned diseases is exceedingly rare. Here, we present a 55-year-old man referred with progressive generalized icterus accompanied by loss of appetite and significant weight loss. The presented case was not an appropriate candidate for liver biopsy due to recent coronary angioplasty and the urgent need for dual antiplatelet therapy. However, medical follow-ups were highly suggestive of concomitant WD, hereditary hemochromatosis, and AIH. The attempts failed for the treatment of hereditary hemochromatosis and WD with chelating agents until the completion of the course of treatment with immunosuppressants targeting components of the AIH-related immune system.

## Introduction

 Besides the coexistence of various immune-related liver diseases, the simultaneous occurrence of hemochromatosis with chronic liver diseases or acute liver failure has also been reported. The iron overload following extensive liver damage is usually secondary to the accumulation of necrotic cells in the hepatic macrophages and is rarely related to HFE (hereditary hemochromatosis gene) mutations, as seen in hepatitis C and non-alcoholic steatohepatitis.^1^ Hemochromatosis is applied to the accumulation and deposition of extra iron in multiple organs, including the heart, liver, pancreas, skin, and gonads, leading to cardiomyopathy, liver failure, diabetes, bronze discoloration of skin, and hypogonadism, respectively. Primary hemochromatosis or hereditary hemochromatosis (HH) originates from HFE gene mutation or other rare genetic disorders leading to excess absorption of iron from the gastrointestinal tract due to the absence or dysfunction of hepcidin or other iron regulatory products. Secondary hemochromatosis is related to iron-producing disorders like ineffective erythropoiesis, liver damage, etc.^2^ They are different in such a way that primary hemochromatosis is associated with parenchymal accumulation of iron and secondary hemochromatosis is predominantly involve reticuloendothelial cells. Literally, high levels of serum iron and transferrin saturation are commonly reported in the setting of acute liver failure or chronic liver disease, but the presence of hereditary hemochromatosis-related mutations as indicators of primary hemochromatosis is exceedingly rare. A high serum iron profile in Wilson’s disease (WD) is justifiable regarding the ferroxidase activity of ceruloplasmin, which transforms ferrous ions into ferric ions. However, this kind of iron overload is not originating from HFE or other related mutations.^3^ WD is a genetic disorder characterized by mutations in ATP7B gene leading to the secretion of copper into the biliary system and its accumulation in the liver, brain, and other organs, as well as the reduction of circulating ceruloplasmin.^4,5^ Copper deposition in organs contributes to an increased risk of neurological and psychological disorders as well as life-threatening liver failure if left undiagnosed and untreated.^6,7^ Absence of serum copper as a key element for the ceruloplasmin ferroxidation activity leads to the accumulation of ferrous ions instead of ferric ions in the bloodstream. Ferrous ions are not transportable to transferrin. Resultantly, they increase iron stores.^8,9^ Autoimmune hepatitis (AIH) is a chronic liver disease in which the immune system invades the hepatocytes, leading to a typical interface hepatitis predominantly infiltrated by lymphocytes and plasma cells in portal tracts extending from zone 1 to zone 3, emperipolesis, and hepatic rosette formation. It constitutes a wide spectrum of clinical presentations from an asymptomatic abnormal liver function test up to a slowly progressive chronic liver disease, and finally, an acute fulminant hepatitis leading to liver failure.^10,11^ Young females are mainly involved, but men, children, and old age are not exempt from the risk.^12^ In the majority of AIH cases, ferritin level is increased as ferritin is believed to be an acute phase reactant. Despite a severe increase in the serum level of ferritin, transferrin saturation and iron stores are not changed. The exact elevation of iron stores as well as the genetic evaluation corroborating HH are extremely rare.^13^ Herein, we present a challenging case of acute hepatitis mimicking features of AIH, WD, and HH while the liver biopsy was contraindicated due to the urgent need for the consumption of dual antiplatelet agents.

## Case Report

**Figure 1:**
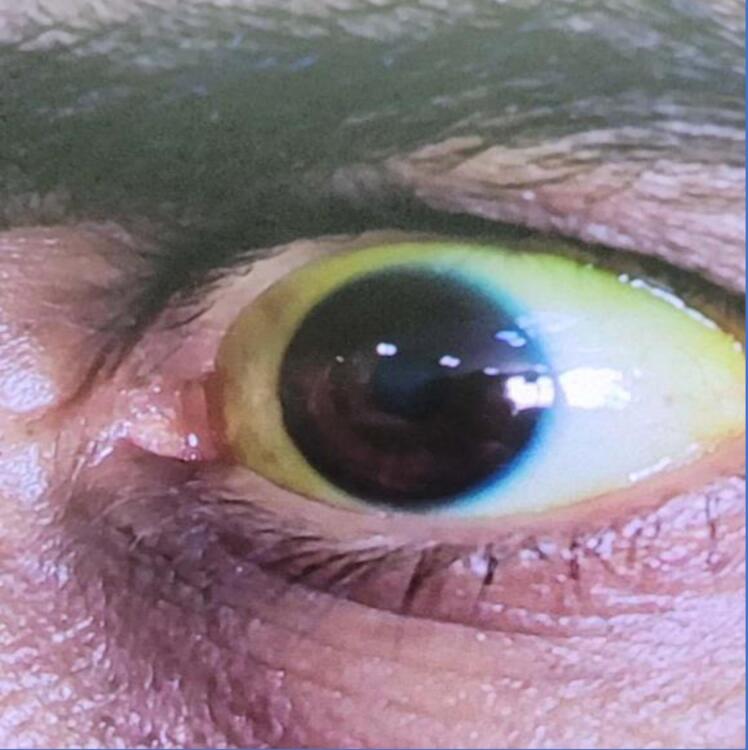
Kayser-Fleischer rings are seen encircling the patient’s cornea

 A 55-year-old man was admitted to Taleghani hospital, a tertiary referral center for liver failure and liver transplantation in Tehran, Iran, with the complaint of a progressive generalized icterus from 2 weeks earlier. His medical history was positive for percutaneous coronary intervention (PCI) and drug-eluting stent implementation. Accordingly, his medication history was remarkable for once-daily 75 mg Plavix and once-daily 80 mg aspirin. His social habit history, travel history, herbal medicine, and over-the-counter drug history were all negative through the last 6 months. Primarily, he was found to have a loss of appetite, leading to a weight loss of about 5 kg in 2 weeks, tea-colored urine, and pale stool. Then, his general health continued to deteriorate, and he was referred to Taleghani center. On admission, his physical examination was not notable except for an icteric sclera, icteric skin, and grade-1 hepatic encephalopathy. He did not appear to have any findings in favor of chronic liver disease, including spider angioma, palmar erythema, caput medusa, gynecomastia, and ascites. Primary laboratory tests presented a direct hyperbilirubinemia [Total: 21 (normal range: 0.1-1.2 mg/dL), Direct: 15 (normal range: < 0.3 mg/dL)], normal alkaline phosphatase [ALP: 89 (normal range: 44-147U/L)], and elevated aminotransferases [AST: 1367 (normal range: 8-33 U/L), ALT: 1706 (normal range: 4-36 U/L)] which raised suspicion of a mixed hepatocellular and cholestatic damage. White blood cells, red blood cells, hemoglobin level, and platelet counts were all within normal range. The standard workup for hemolysis consisted of serum lactate dehydrogenase, reticulocyte count, Coombs test, and haptoglobin test, which was not indicative of hemolysis. Renal function tests, serum electrolytes, serum uric acid, and blood sugar were normal. Prolongation of prothrombin time up to 16 seconds (normal range: 11-13.5 seconds) as well as some degrees of covert encephalopathy, raised suspicion of liver failure in the presented case. Serum iron profile revealed high level of ferritin [approximately 6587 (normal range: 24-336 µg/L)] and high level of transferrin saturation [approximately 111% (normal range: 20-50%) with serum iron level of about 258 (normal range: 60-170 mcg/dL), and total iron binding capacity (TIBC) of about 232 (normal range: 240-450 mcg/dL)] consistent with hemochromatosis. Secondary workups were followed by a complete panel test of viral hepatitis, including hepatitis A virus (HAV), hepatitis B virus (HBV), hepatitis C virus (HCV), Epstein-Barr virus (EBV), cytomegalovirus (CMV), and herpes simplex virus (HSV), which were negative. Autoantibody serology tests looking for AIH and overlap syndromes, including anti nuclear antibody (ANA), anti-smooth muscle antibody (ASMA), anti-liver kidney microsomal (Anti-LKM), and anti-mitochondrial antibody (AMA), were positive for ANA (1/40), ASMA (1/80), and anti-LKM1 (1/80). Serum protein electrophoresis revealed normal serum albumin level and polyclonal hypergammaglobulinemia indicative of the absence of previous liver involvement. Laboratory tests to rule out WD revealed a significantly decreased serum ceruloplasmin [up to 2 (normal range: 14-40 mg/dL)] and 24-hour urine copper measuring about 129 (normal range: 20-50 µg/24 h). As we know, urine copper can be increased during the course of acute hepatitis and cholestatic diseases, the 24-hour urine copper test was also repeated after patient discharge, which was about 98 (normal range: 20-50 µg/24 h). Abdominopelvic ultrasonography using color Doppler ultrasound revealed a normal-appearing abdomen. Given the results, differential diagnosis lies among primary or secondary hemochromatosis, WD, and AIH. More advanced tests were requested to narrow down the list of differential diagnoses. In terms of hemochromatosis, serum ferritin levels suffer from a relatively poor specificity as they increase extremely high during the course of severe diseases as an acute phase reactant. Normal physical examination, normal liver magnetic resonance imaging (MRI), and absence of other organ involvement were against the diagnosis of a previously established hemochromatosis. Contradictory results led to genetic consultation and genetic testing for hereditary hemochromatosis, which finally revealed a homozygous mutation in HFE genes. As seen with high levels of serum ferritin, a significant decrease in the level of serum ceruloplasmin was suggestive of an acute phase reactant in the course of severe disease, so it was not specific for the diagnosis of WD. However, an increased level of 24-hour urine, which was repeated after discharge of the patient, and the presence of a Kayser-Fleischer ring using a slit-lamp in the anterior chamber of the patient’s eye (Figure 1) confirmed the diagnosis of WD without the need for further investigation. To elaborate the accuracy of diagnosis, the Leipzig probability scoring test was also calculated for the patient, which revealed a score of 6, including 2 points for Kayser-Fleischer rings, 2 points for 24-hour urine copper > 2 times the upper limit of normal, 2 points for serum ceruloplasmin level < 10 mg/dL. As evidenced, a score ≥ 4 is considered to be highly probable for WD. Significant levels of autoantibodies were suggestive but not diagnostic for AIH. Therefore, we decided to proceed with a liver biopsy. However, the history of recent PCI leading to dual antiplatelet therapy was a contraindication for diagnostic percutaneous liver biopsy since the cardiologist was against the discontinuation of antiplatelet agents. As a result, the diagnosis of AIH was a real challenge in this case. Lack of liver histological evaluation in addition to a probability score of 15 including -3 points for AST level > 3 times the upper limit of normal, 3 points for IgG level > 2 times the upper limit of normal, 3 points for high titers of ANA and ASMA, 3 points for negative viral markers, 1 point for no consumption of hepatotoxic drugs, and 2 points for no consumption of alcohol revealed a probable AIH in the presented case. As driven by the laboratory tests, the diagnostic criteria for HH and WD were complete. Regarding the fact that liver biopsy was impossible, he was started on D-penicillamine 250 mg twice daily, zinc sulfate 50 mg thrice daily, and phlebotomy twice weekly, which were not promising till the initiation of standard treatment of AIH, including prednisolone 1mg/kg daily which resulted in a dramatic response in serum bilirubin level. Then, the dose of prednisolone was slowly tapered up to 10 mg/d. To reduce steroid dosage into the lowest probable dose, azathioprine1mg/kg daily was added to the regimen. The post-treatment score of the patient was 17, which shows a definite diagnosis of AIH based on the diagnostic scoring system of the international AIH group.

 Eventually, serum bilirubin level declined to near normal over a 2-month trial of treatment. Maintenance therapy following a tapering dose of prednisolone in addition to azathioprine and chelating agents was continued.

## Discussion

 A step-by-step approach to diagnosis of the underlying cause of acute liver failure was performed. The clinical scenario was suggestive of a flare-up of an already-existent hepatic disorder after PCI. A literature review revealed no clear association between angioplasty, contrast media injection, antiplatelet consumption, and acute liver injury. The relation between liver failure and cardiac disease is clearly defined in patients with right-sided heart failure, leading to hepatic congestion and ischemic hepatitis.^14^ Among cardiovascular drugs, statins were reported to be associated with a wide spectrum of drug-induced liver injuries from an asymptomatic elevation in aminotransferases, acute hepatocellular hepatitis, acute cholestatic hepatitis to autoantibody-associated drug-induced liver injury.^15^ The patient’s medication history was negative except for antiplatelet agents, which are not reported to be related to liver injury, literally. Acute hepatitis has a wide variety of etiologies. The timely and definite diagnosis and treatment of an acute liver injury is of paramount importance. Hemochromatosis, especially HH, is not a probable diagnosis in cases without a positive family history of hemochromatosis, without a positive personal history of underlying disorders leading to an increase in the iron stores, and without any evidence of other organ involvement, including cardiomyopathy, diabetes, skin hyperpigmentation, and hypogonadism. Although many chronic liver diseases such as non-alcoholic steatohepatitis (NASH), chronic hepatitis B and C, primary biliary cholestasis, and alpha-1 antitrypsin deficiency are associated with mild iron overload and near-normal transferrin saturation rarely exceeding 45%, the association between primary & secondary hemochromatosis and other liver diseases like AIH is exceedingly rare.^1^ The natural history of chronic liver diseases with a genetic mutation in HFE gene family, even in the absence of iron overload, is reported to be more progressive into liver fibrosis and cirrhosis.^1^ Consequently, it would be a trigger factor for the earlier presentation of underlying chronic liver diseases, including AIH and WD, in a patient with a positive balance of iron absorption from the gastrointestinal tract. Given the transferrin saturation of about 110% following a genetic study indicative of a homozygous variant of H63D in the HFE gene family, the diagnosis of HH was confirmed for the patient. However, the acute presentation of liver failure was not attributed to iron stores as the patient was non-responsive to phlebotomy and was not found to have other organ involvements suggestive of hemochromatosis. There are some rare reports worldwide indicative of the coexistence of acute and chronic liver diseases, as seen in the case report of Warner and colleagues, which shows the coexistence of HH and AIH in a 57-year-old man^16^ and the case report of Glass and Dickson who presented a rare case of HH, WD, and alpha-1-antitrypsin deficiency.^17^ In terms of WD, the Leipzig scoring criteria for diagnosis was 6, which is suggestive of an established diagnosis of WD. Based on the criteria, serum ceruloplasmin level less than 0.1 g/L, 24-hour urine copper higher than 2 times the upper limit of normal, and the presence of a Kayser-Fleischer ring led to a score of 6, total.^18^ The coexistence of WD and secondary hemochromatosis is obvious due to the fact that the transport of iron ions is disrupted in the course of WD due to low levels of ceruloplasmin, as what is seen in aceruloplasminemia.^19^ However, non-specific brain MRI for end-stage WD, as well as patient non-response to D-penicillamine, raised suspicion of another reason for liver failure in the presented case. Simultaneous presentation of WD and AIH has been rarely reported.^20^ Regarding the diagnosis of AIH, care should be taken that there are a lot of other causes of liver failure mimicking AIH. They should be ruled out before considering AIH as a definite diagnosis of liver failure. As could be seen in the case report of Santos and others, there was a 25-year-old woman who presented with signs and symptoms of chronic liver disease with elevated levels of autoantibodies and gamma globulins, mimicking AIH non-responsive to immunosuppression. Finally, further evaluations revealed the presence of WD as a mimicker of AIH.^21^ One of the other important underlying causes that should be ruled out is autoimmune-related drug-induced liver injury. As mentioned before, our patient’s drug history was unremarkable except for antiplatelet agents. The gold standard of AIH for diagnosis and treatment is liver biopsy; however, it was not possible for the presented case as he was on dual antiplatelet therapy since a month earlier. A high serum ferritin level as an acute phase reactant is seen during the course of AIH. However, elevated serum ferritin levels following the elevated level of transferrin saturation are not common.^22^ Given the aggregate score of 15 based on the international AIH group scoring system, the patient was highly likely to have AIH. The patient was started on prednisolone and then azathioprine and prednisolone. He dramatically responded to immunosuppressive therapy. Serum aminotransferases and bilirubin levels were all within normal ranges 6 months from initiation of immunosuppressive therapy. The patient continued to remain stable after that. There are several cases of WD patients, who present superimposed manifestations of AIH.^23,24^ These patients are candidates for combination therapy with penicillamine and steroids, as is what was followed in the presented case. Herein, we reported a diagnostic challenge in a case of acute liver failure, which was found to have a coexistence of 3 lab-confirmed liver diseases, including HH, WD, and AIH. To confirm the definite diagnosis, liver biopsy was not attempted due to the increased risk of bleeding on dual antiplatelet therapy. The patient did not respond to either of the trial therapies for HH and WD, but the response to the standard treatment of AIH based on prednisolone and then azathioprine plus prednisolone was dramatic.

## Conclusion

 Although rare in the literature, the coexistence of WD, AIH, and HH is anticipated. If the real reason for liver failure was not determined, simultaneous therapy of the three labeled diseases would be reasonable.
